# Socioeconomic status impacts tumor biology, treatment, and outcomes in over 200,000 patients with invasive lobular carcinoma of the breast: an analysis of the National Cancer Database

**DOI:** 10.1007/s10549-025-07769-5

**Published:** 2025-06-26

**Authors:** Mandeep Kaur, Astrid Quirarte, Amy M. Shui, Anna Vertido, Elle Clelland, Harriet Rothschild, Laura J. Esserman, Cheryl Ewing, Rita A. Mukhtar

**Affiliations:** 1 School of Medicine, University of California, San Francisco, CA USA; 2 Division of Surgical Oncology, Department of Surgery, University of California, 1825 4Th Street, 3Rd Floor, Box 1710, San Francisco, CA 94143 USA; 3 Department of Epidemiology and Biostatistics, University of California, San Francisco, CA USA

**Keywords:** Invasive lobular carcinoma, Socioeconomic status, Insurance, Breast cancer outcomes

## Abstract

**Purpose:**

While the impact of socioeconomic factors on breast cancer diagnosis, treatment, and outcomes are well-documented, few studies have focused on invasive lobular carcinoma (ILC), the second most common type of breast cancer. We evaluated the relationships between race and socioeconomic status (SES) with clinicopathological characteristics and outcomes in patients with stage I-III ILC using the National Cancer Database (NCDB).

**Methods:**

We used the NCDB, a national oncology database, to evaluate insurance status, a composite measure of SES (education and income), clinicopathological characteristics, and outcomes in patients with stage I-III ILC. Clinicopathologic variables included tumor size, presence of lymphovascular invasion (LVI), and tumor receptor subtype (hormone receptor, HR), and tumor grade. Overall survival was analyzed with multivariable Cox proportional hazards models.

**Results:**

We identified 269,657 patients with stage I-III ILC. Patients in the Medicaid/no insurance group and those with lower SES had larger tumors, more positive lymph nodes, fewer HR+ tumors, and higher-grade tumors. Those in the low SES group had higher rates of chemotherapy use and, in those with HR+ tumors, lower rates of endocrine therapy use. In a multivariable model adjusting for SES, self-identified race/ethnicity, age, stage, receptor subtype, grade, treatment, and Charlson–Deyo score, patients with low SES had a 24% higher risk of death by 5 years compared to patients with high SES (HR 1.24, 95% CI 1.19–1.30, p < 0.001).

**Conclusion:**

While our study confirms several known disparities in the presentation, outcomes, and treatment of breast cancer, this is the first evaluation to assess how different components of SES influence ILC specifically.

## Introduction

Breast cancer is a leading cause of cancer-related deaths in women across the globe [1]. Invasive lobular carcinoma (ILC) is the second most common type of breast cancer, accounting for up to 15% of all breast cancer cases [2]. Compared to the more prevalent invasive ductal carcinoma (IDC), ILC is characteristically more hormonally driven and takes on a diffuse, infiltrative pattern of growth due to the lack of E-cadherin [2]. This contributes to its distinct appearance on imaging, response to systemic therapy, and pattern of recurrence, all of which distinguish ILC from IDC [3, 4]. Recent investigations have shown strong correlations between differences in socioeconomic status (SES) and variations in breast tumor features, treatment, and survival, indicating a potentially critical role of SES in breast cancer outcomes, but have not focused on differences by tumor histology [5–8].

Socioeconomic status (SES) is a composite measure of an individual’s economic or social position relative to others, typically including measures like education and income, and has well-established associations with various health outcomes, including cancer [9–14]. In a single institution analysis of patients with ILC, we previously established that lower SES, as measured by the Area Deprivation Index (ADI), is linked to larger tumors, increased rates of lymphovascular invasion (LVI), and higher grade tumors [15]. Additionally, we found lower SES to be associated with higher likelihood of mastectomy and lower receipt of endocrine therapy, highlighting discrepancies in treatment related to variations in SES. Other studies evaluating health disparities in patients with ILC have shown higher rates of diagnosis and worse outcomes for those with non-White race [16, 17]. To date, these investigations examining the relationship between socioeconomic factors and ILC remain limited.

While associations between SES and breast cancer in general have been well-established, these findings may not fully translate to ILC due to its distinct biological and clinical characteristics. To address this, we queried the National Cancer Database (NCDB) to assess the relationships between a composite measure of SES, using income and education, and the clinicopathological features, treatment, and outcomes of a large cohort of patients with stage I-III ILC. Additionally, we evaluated whether prior associations between race and outcomes in those with ILC would persist when considering composite measures of SES.

## Methods

This was a retrospective cohort study using the 2010–2016 National Cancer Database (NCDB) participant user files (PUFs). The National Cancer Database is a comprehensive database containing data from over 1,500 sites across the United States, capturing approximately 70% of all newly diagnosed cancer cases in the United States [18]. We collected socioeconomic status, clinicopathologic characteristics, and survival outcomes for patients with stage I-III ILC treated at all NCDB-participating institutions. Patients with stage IV or metastatic disease were excluded.

Covariates were selected based on clinical relevance and their potential role as confounders in the relationship between SES and ILC presentation, treatment, and outcomes. Race and ethnicity data were collected and grouped as White-identifying, Black-identifying, East Asian-identifying (including Chinese, Japanese, and Korean), Spanish/Hispanic origin-identifying (as defined by the NCDB), or Other. Tumor receptor subtype was classified by estrogen receptor (ER), progesterone receptor (PR), and human epidermal growth factor 2 (HER2) status, and grouped as HR+HER2−, HR−HER2− (triple negative), or HER2+. To capture a broader measure of SES, we created a composite SES score using annual income and education quartiles. For income, the NCDB grouped annual income into the following categories: < $40,227, $40,227–$50,353, $50,354–$63,332, and ≥ $63,333. The NCDB’s education quartiles were defined by the following percentages of people over the age of 25 in the patient’s zip code without a high school degree: ≥ 17.6%, 10.9%–17.5%, 6.3%–10.8%, and < 6.3%. These quartile assignments (1, 2, 3, 4) were then added together to create a composite measure of SES, which was grouped as follows: low (2–3), mid-low (4–5), mid-high (6–7), and high (8) [19]. Insurance status, as a categorical variable, was collected and analyzed separately. Categories were private insurance, Medicare, and Medicaid/no insurance. Given the small proportion of uninsured patients (< 1%), and the expansion of Medicaid coverage to uninsured patients under the Breast and Cervical Cancer Prevention and Treatment Act, we combined the Medicaid and no insurance. We excluded the < 3% of subjects with “other government insurance” and unknown insurance. Comorbidities were assessed using the Charlson–Deyo Comorbidity Index, ranging from 0 to 6 with 0 representing no comorbid health conditions, 1 and 2 representing mild comorbid health conditions, and ≥ 3 representing moderate to severe comorbidities [20].

### Statistical analysis

Data were analyzed in Stata 16.1. Hypothesis tests were two-sided, and the significance threshold was set to 0.05. Chi-squared tests were used to compare categorical variables and analysis of variance (ANOVA) to compare continuous variables across groups. We compared tumor characteristics (size, number of positive lymph nodes, receptor subtype, grade, and LVI) and treatment type (receipt of chemotherapy, endocrine therapy, and type of surgery) by insurance type and composite SES (based on income and education quartiles) in patients with stage I-III ILC. We also used logistic regression to evaluate the association between socioeconomic status and surgery type, and we used multinomial logistic regression to evaluate the association between socioeconomic status and receptor subtype while controlling for race/ethnicity.

We evaluated overall survival using a Cox proportional hazards model for insurance type and composite SES, controlling for self-identified race/ethnicity, age at diagnosis, stage, receptor subtype, tumor grade, receipt of chemotherapy, receipt of endocrine therapy, and Charlson–Deyo comorbidity score. We performed a sensitivity analysis adding distance from hospital and urban/metro vs. rural (2013 classification) to the model. Administrative censoring was applied at five years. We analyzed the unknown/missing category for variables that had > 5% missing data and used complete case analysis for all other variables.

## Results

### Study cohort

We identified 269,657 cases of patients with stage I-III ILC in the NCDB. Of these, 226,868 identified as White (84%), 20,948 as Black (7.8%), 11,848 as Spanish/Hispanic (4.4%), 1,817 as East Asian (0.7%), and 5,889 as other (2.2%). Overall, most tumors (92.4%) were HR+HER2−, consistent with prior studies of ILC. Most tumors were grade 2, and LVI was present in 15%. Among those with HR+ tumors (92.4%), 88% received endocrine therapy. Chemotherapy was utilized in 38% of the entire cohort. For surgical treatment, 51% underwent mastectomy and 49% underwent lumpectomy (see Table 1).

**Table 1:** ILC characteristics in overall study cohort

Variable	Study cohort (N = 269,657)
Age, years (mean ± SD)	62.9 ± 12.4
Self-identified race/ethnicity	
White	84%
Black	7.8%
East Asian	< 1%
Spanish/Hispanic	4.4%
Other	2.2%
[unknown/missing, n]	[2287]
Income	
< $40,227	13%
$40,227–$50,353	19%
$50,354–$63,332	23%
≥ $63,333	45%
[unknown/missing, n]	[3816]
Education (no HS degree)	
≥ 17.6%	15%
10.9%–17.5%	22%
6.3%–10.8%	29%
< 6.3%	33%
[unknown/missing, n]	[3383]
Tumor size, mm (mean ± SD)^a^	25.8 ± 35.3
Positive lymph nodes, n (mean ± SD)^b^	1.64 $$\pm$$ 4.14
Receptor Subtype	
HR+/HER2−	92.4%
HR−/HER2−	1.8%
HER2+ [unknown/missing, n]	5.8% [117,577]
Tumor grade	
1	26%
2	62%
3 [unknown/missing, n]	12% [26,968]
Lymphovascular invasion present [not applicable, n] [unknown/missing, n]	15% [6] [21,569]
Endocrine therapy* [unknown/missing, n]	88% [3215]
Chemotherapy [unknown/missing, n]	38% [8181]
Surgery	
Mastectomy	51%
Lumpectomy [unknown/missing, n]	49% [1150]

Data reported from complete case analyses. SD, standard deviation; HR, hormone receptor; HER2, human epidermal growth factor receptor 2; +  receptor status positive; − receptor status negative. *Endocrine therapy in HR+ patients only (n = 140,585)

^a^n = 267,109

^b^n = 257,993

### Insurance type

Most patients had private insurance (52%), followed by Medicare (42%) and Medicaid/no insurance (5.8%). Patients with Medicare were older than patients with private insurance and Medicaid/no insurance (73.2 ± 8.0 years compared to 55.7 ± 9.4 years for private insurance and 54.7 ± 10.1 years for those with Medicaid/no insurance). Insurance differed significantly by self-identified race/ethnicity (p < 0.001), with a smaller proportion of Black-identifying and Spanish/Hispanic-identifying patients having private insurance (48% and 48%, respectively, versus 52% for White, 65% for East Asian, and 61% for Other). Those with Medicaid/no insurance had larger tumors, more positive lymph nodes, more triple negative tumors, more grade 3 tumors, and more LVI (Table 2). Receipt of chemotherapy and endocrine therapy differed significantly by insurance type (both p < 0.001). Those with Medicaid/no insurance received chemotherapy more often than those with private insurance or Medicare (53% versus 48% in private insurance and 23% in Medicare insurance groups). Patients with private insurance received endocrine therapy more commonly (84% versus 81% in Medicaid/no insurance group and 78% in Medicare group). There were no clinically significant differences in receipt of surgery by insurance status (99.7% vs. 99.7% vs. 99.5% for private, Medicare, and Medicaid/no insurance, respectively), however, the type of surgery received differed (p < 0.001). Patients with Medicare underwent lumpectomy more frequently (52% in Medicare insurance group versus 46% in private insurance group and 42% in Medicaid/no insurance group).

**Table 2:** ILC characteristics by insurance

Characteristic	Insurance (n = 262,463)			
	Medicaid/no insurance (n = 15,303)	Medicare (n = 110,831)	Private insurance (n = 136,329)	p-value
Age, years (mean ± SD)	54.7 ± 10.1	73.2 ± 8.0	55.7 ± 9.4	< 0.001
Tumor size, mm (mean ± SD)^a^	30.0 ± 38.5	24.2 ± 26.9	25.0 ± 30.7	< 0.001
Positive lymph Nodes, n (mean ± SD^)b^	2.4 $\pm$ 5.0	1.5 $\pm$ 4.1	1.7 $\pm$ 4.1	< 0.001
Receptor subtype				< 0.001
ER+/PR+HER2−	49%	46%	46%	
ER+/PR−/HER2−	5.7%	8.1%	5.0%	
HER2+	4.3%	3.1%	3.3%	
Triple negative	1.3%	1.1%	< 1%	
Unknown/missing	40%	42%	45%	
Tumor grade				< 0.001
1	21%	25%	23%	
2	55%	55%	55%	
3 Unknown/missing	15% 10%	10% 9.9%	11% 10%	
Lymphovascular invasion Not present Unknown/missing [not applicable, n]	18% 68% 15% [0]	12% 75% 13% [5]	14% 73% 13% [0]	< 0.001

Data reported from complete case analyses. Unknown/missing category analyzed if data missing for > 5%. SD, standard deviation; HR, hormone receptor; HER2, human epidermal growth factor receptor 2; +  receptor status positive; − receptor status negative

^a^n = 260,089

^b^n = 251,629

### Composite socioeconomic status

When grouped by composite SES, 43,394 (16%) patients were categorized as having low SES, 65,472 (25%) had mid-low SES, 84,908 (32%) had mid-high SES, and 72,067 (27%) had high SES. Composite SES category differed significantly by self-identified race (p < 0.001), with Black-identifying patients having the smallest proportion of patients with high SES and the greatest proportion with low SES (8.9% with high SES versus 29% for White, 38% for East Asian, 12% for Spanish/Hispanic, and 30% for Other; 45% with low SES versus 13% for White, 9.5% for East Asian, 36% Spanish/Hispanic, and 12% for Other). Similar to patients in the Medicaid/no insurance group, those with lower SES had larger tumors, more positive lymph nodes, fewer HR positive tumors, more triple negative tumors, more grade 3 tumors, and more LVI (Table 3). While triple negative and HER2 + tumors were rare in this dataset, we found a significant association between receptor subtype and composite SES category (p < 0.001). Higher rates of triple negative ILC in those with lower composite SES (1.3% triple negative in low SES group and < 1% in high SES group). This association persisted when controlling for race/ethnicity, with patients with low SES more likely to have triple negative subtype. Compared to the high SES group, those with low SES had 1.35 times the odds of triple negative subtype (OR 1.35, 95% CI 1.20–1.53, p < 0.001). There were small but statistically significant differences in systemic therapy by composite SES category. Those in the low SES group had the highest proportion receiving chemotherapy and, for those with HR+ tumors, the lowest proportion receiving endocrine therapy (33% chemotherapy in low SES versus 30% for high SES; 88% endocrine therapy in the low SES group versus 89% for high SES when restricted to HR+ subtypes). There were no clinically significant differences in the receipt of surgery overall by SES (99.4% vs. 99.6% vs. 99.6% vs. 99.6% for low, mid-low, mid-high, and high SES, respectively). However, the odds of mastectomy increased by 7% as for each category decrease in SES (OR 1.07, 95% CI 1.06–1.08, p < 0.001: 50% had mastectomy in the high SES group, 51% in the mid-high group, 53% in the mid-low group, and 55% in the low SES group).

**Table 3:** ILC characteristics by composite SES

	Composite SES (n = 265,841)				
Characteristic	Low (n = 43,394)	Mid-low (n = 65,472)	Mid-high (n = 84,908)	High (n = 72,067)	p-value
Tumor size, mm (mean ± SD)^a^	27.3 ± 36.7	26.6 ± 36.1	25.4 ± 34.3	24.6 ± 34.8	< 0.001
Positive lymph Nodes, n (mean ± SD)^b^	1.9 $\pm$ 4.4	1.8 $\pm$ 4.3	1.6 $\pm$ 4.1	1.5 $\pm$ 3.9	< 0.001
Receptor subtype					< 0.001
ER+/PR+/HER2-	45%	45%	46%	47%	
ER+/PR−/HER2-	6.5%	6.3%	6.4%	6.3%	
HER2+	3.6%	3.4%	3.2%	3.0%	
Triple negative	1.3%	1.0%	< 1%	< 1%	
Unknown/missing	44%	44%	43%	43%	
Tumor grade					< 0.001
1	23%	24%	23%	24%	
2	54%	55%	56%	56%	
3 Unknown/missing	12% 11%	11% 10%	11% 9.9%	10% 9.5%	
Lymphovascular invasion Not present Unknown/missing [not applicable, n]	14% 70% 15% [1] [1]	13% 72% 14% [3] [3]	13% 73% 13% [1] [1]	13% 75% 12% [1] [1]	< 0.001

Data reported from complete case analyses. Unknown/missing category analyzed if data missing for > 5%. SD, standard deviation; HR, hormone receptor; HER2, human epidermal growth factor receptor 2; +  receptor status positive; − receptor status negative

^a^n = 263,337

^b^n = 254,341

### Overall survival

In a multivariable model adjusted for insurance type, composite SES, age at diagnosis, stage, receptor subtype, tumor grade, receipt of chemotherapy, receipt of endocrine therapy, and Charlson–Deyo comorbidity score, Black-identifying participants had a 15% higher risk of death at 5 years after diagnosis compared to White-identifying participants (Table 4). In contrast, East Asian-identifying, Spanish/Hispanic-identifying, and Other participants had a 21%, 27%, and 28% lower risk of death at 5 years compared to White-identifying patients, respectively. Other factors associated with improved overall survival in this model included receipt of chemotherapy and endocrine therapy. Medicare or Medicaid/no insurance was associated with shorter overall survival compared to private insurance. Lower SES was associated with shorter overall survival compared to high SES, with the low SES group having 24% increased risk of death. Higher stage disease, ER+/PR-/HER2- or triple negative subtype compared to ER+/PR+/HER2− subtype, higher grade tumors, and higher Charlson-Deyo comorbidity score were also associated with shorter overall survival.

**Table 4:** Multivariable cox proportional hazards model for death by five years with insurance status, composite socioeconomic status, self-identified race/ethnicity, age at diagnosis, stage, grade, receptor subtype, receipt of chemotherapy, receipt of endocrine therapy, and Charlson–Deyo comorbidity index in patients with ILC

Variables	Hazard ratio	95% confidence interval	p-value
Private insurance (reference)			
Medicare insurance	1.20	1.15–1.24	< 0.001
Medicaid/no insurance			
High SES (reference)	1.75	1.65–1.86	< 0.001
Low SES	1.24	1.19–1.30	< 0.001
Mid low SES	1.23	1.19–1.28	< 0.001
Mid high SES	1.11	1.07–1.15	< 0.001
White (reference)			
East Asian	0.79	0.64–0.99	0.04
Black	1.15	1.10–1.21	< 0.001
Spanish/Hispanic	0.73	0.68–0.79	< 0.001
Other	0.72	0.63–0.82	< 0.001
Age at Diagnosis (per year)	1.05	1.05–1.05	< 0.001
Stage 1 (reference)			
Stage 2	1.66	1.60–1.72	< 0.001
Stage 3	4.38	4.22–4.55	< 0.001
Grade 1(reference)			
Grade 2	1.14	1.10–1.18	< 0.001
Grade 3 Unknown/missing	1.69 1.18	1.61–1.76 1.12–1.24	< 0.001 < 0.001
ER+/PR+/HER2− (reference)			
ER+/PR−/HER2−	1.43	1.35–1.51	< 0.001
Triple negative	1.68	1.53–1.85	< 0.001
HER2+ Unknown/missing	1.11 1.11	1.01–1.21 1.07–1,14	0.02 < 0.001
Receipt of Chemotherapy	0.83	0.80–0.86	< 0.001
Receipt of Endocrine Therapy	0.49	0.47–0.50	< 0.001
Charlson Deyo Score 0 (reference)			
Charlson Deyo Score 1	1.47	1.42–1.52	< 0.001
Charlson Deyo Score 2	2.25	2.13–2.39	< 0.001
Charlson Deyo Score $\ge$ 3	3.07	2.80–3.36	< 0.001

Model included insurance, composite SES, race/ethnicity, age, stage, receptor subtype, grade, receipt of chemotherapy, receipt of endocrine therapy and Charlson–Deyo comorbidity index score, *ER* estrogen receptor, *PR* progesterone receptor, *HER2* human epidermal growth factor receptor 2; +  receptor status positive; − receptor status negative. Data available in n = 219,316

When adding distance from hospital and urban/metro versus rural classification to the above multivariable model, urban/metro versus rural classification was not significantly associated with overall survival (HR 1.01, 95% CI 0.91–1.13, p = 0.82), and all other associations reported from the main model remained almost identical in magnitude, direction, and significance.

## Discussion

Our study demonstrates that known disparities in breast cancer treatment and outcomes extend to those with ILC, the second most common type of breast cancer. Although a majority of ILC tumors were HR+/HER2− and low to intermediate grade, we found that low SES was associated with more aggressive tumor features, including higher grade and increased LVI. These findings are consistent with our prior single-institution work where we found that greater area deprivation index was associated with larger ILC tumor size and greater LVI [15]. We also observed similar differences in treatment in both this national analysis and our institutional cohort, with lower SES being associated with higher mastectomy rates and less endocrine therapy use in those with HR+ disease. The consistency between our single-institution findings and this national cohort strengthens the evidence that socioeconomic factors impact ILC presentation and outcomes.

These findings are also in line with recent advancements in our understanding of ILC. Although ILC has historically been viewed as a homogenous tumor type, recent studies show heterogeneity in the molecular subtypes of ILC [21–23]. The presence of high grade and LVI in ILC is atypical, however we found that these factors were significantly more common in those with lower SES suggesting that tumor heterogeneity in ILC may be influenced by SES factors. The underlying mechanism driving these aggressive features in a typically low proliferative tumor type is unknown and remains an important area for future research.

Recent studies have shown that outcomes disparities by self-identified race appear to impact those with HR positive breast cancers in particular. Black-identifying patients with ILC experienced worse overall survival compared to other race groups, despite the generally favorable prognosis associated with HR+ tumors. This trend is supported by existing literature that has identified similarly worse survival and outcomes for Black-identifying patients with HR+ tumors [24–26]. Proposed explanations for these disparities include differences in tumor biology, treatment delays, and barriers to consistent healthcare [27, 28]. Additionally, the impact of an individual’s lived environment may have significant impact on tumor biology. Persistent social, political, and economic marginalization of Black-identifying women may contribute to higher comorbidities, changes in allostatic load, and poorer outcomes [29–31]. This concept, coined the weathering hypothesis, has been supported by several studies as a possible explanation for how environmental challenges may contribute to epigenetic changes in Black-identifying patients [32–34]. Investigators have shown that chronic stress from neighborhood disadvantage is associated with the upregulation of pro-inflammatory pathways, such as the Conserved Transcriptional Response to Adversity (CTRA) gene expression profile in leukocytes [35–38]. The CTRA response has been shown to be increased in both low-income and Black-identifying patients, offering a potential biologic mechanism through which structural inequities influence clinical outcomes [39–42].

In our analysis, the observation of high stage at presentation could be partially explained by limited access to screening mammography in those with lower SES [43]. Educational levels and health literacy, for example, may also impact patients’ understandings of breast cancer symptoms and screening guidelines, influencing how they engage with the healthcare system [44, 45]. Geographic barriers, such as residence in rural or medically underserved areas, can also limit access to timely care, especially when coupled with transportation challenges and caregiving or work responsibilities [46, 47]. Additionally, the use of hormone replacement therapy—which has been associated with the development of ILC—is more common in those with higher SES [48, 49]. In one analysis, those taking combined estrogen and progestin hormone replacement therapy for menopausal symptoms had significantly increased risk of developing ILC compared to non-users [50].

In summary, our analysis of the NCDB validates our previous single institution findings, showing that socioeconomic factors influence not only tumor biology but also treatment and outcomes in patients with ILC. Limitations include the high prevalence of missing data in the NCDB, and the lack of data on important factors such as surveillance, duration of endocrine therapy, and adherence to endocrine therapy [51]. Our study did not capture breast cancer-specific survival, limiting our ability to distinguish between deaths caused by tumor biology and other causes, including factors associated with SES. Additionally, the diagnosis of ILC was not centrally confirmed, raising the possibility that some histologic diagnoses were incorrectly classified as the diagnostic challenges of ILC are well-documented [52]. Finally, because of the lack of more granular SES data in the NCDB and the inability to use more complex measures of SES, such as the Area Deprivation Index, our reliance on education and income as SES indicators may not fully capture other relevant aspects of SES that could impact outcomes.

These findings highlight the potential for heterogeneity in ILC, and the importance of understanding how socioeconomic factors influence tumor development and outcomes. Our composite measure of SES made use of income and education, variables that have independently been associated with cancer outcomes and are readily available in the NCDB, to better model cumulative socioeconomic disadvantage. Future studies should investigate the mechanisms driving the development of aggressive ILC features in those with low SES, with a focus on the roles of genetics, environment, and access to treatment.

## Data Availability

The National Cancer Data Base (NCDB) is a joint project of the Commission on Cancer (CoC) of the American College of Surgeons and the American Cancer Society. The datasets analyzed during the current study are available in the National Cancer Database, https://www.facs.org/quality-programs/cancer-programs/national-cancer-database/.

## References

[1] Arnold M, Morgan E, Rumgay H et al (2022) Current and future burden of breast cancer: global statistics for 2020 and 2040. Breast Off J Eur Soc Mastol 66:15–23. 10.1016/j.breast.2022.08.010 10.1016/j.breast.2022.08.010 PMC9465273 36084384

[2] McCart Reed AE, Kalinowski L, Simpson PT, Lakhani SR (2021) Invasive lobular carcinoma of the breast: the increasing importance of this special subtype. Breast Cancer Res 23(1):6. 10.1186/s13058-020-01384-6 33413533 10.1186/s13058-020-01384-6 PMC7792208

[3] Thomas M, Kelly ED, Abraham J, Kruse M (2019) Invasive lobular breast cancer: a review of pathogenesis, diagnosis, management, and future directions of early stage disease. Semin Oncol 46(2):121–132. 10.1053/j.seminoncol.2019.03.002 31239068 10.1053/j.seminoncol.2019.03.002

[4] Pramod N, Nigam A, Basree M et al (2021) Comprehensive review of molecular mechanisms and clinical features of invasive lobular cancer. Oncologist 26(6):e943–e953. 10.1002/onco.13734 33641217 10.1002/onco.13734 PMC8176983

[5] Dreyer MS, Nattinger AB, McGinley EL, Pezzin LE (2018) Socioeconomic status and breast cancer treatment. Breast Cancer Res Treat 167(1):1–8. 10.1007/s10549-017-4490-3 28884392 10.1007/s10549-017-4490-3 PMC5790605

[6] Puthanmadhom Narayanan S, Ren D, Oesterreich S, Lee AV, Rosenzweig MQ, Brufsky AM (2023) Effects of socioeconomic status and race on survival and treatment in metastatic breast cancer. Npj Breast Cancer 9(1):1–5. 10.1038/s41523-023-00595-2 37914742 10.1038/s41523-023-00595-2 PMC10620133

[7] Hossain F, Danos D, Prakash O et al (2019) Neighborhood social determinants of triple negative breast cancer. Front Public Health 7:18. 10.3389/fpubh.2019.00018 30834239 10.3389/fpubh.2019.00018 PMC6387917

[8] Akinyemiju TF, Pisu M, Waterbor JW, Altekruse SF (2015) Socioeconomic status and incidence of breast cancer by hormone receptor subtype. Springerplus 4:508. 10.1186/s40064-015-1282-2 26405628 10.1186/s40064-015-1282-2 PMC4573746

[9] Wang J, Geng L (2019) Effects of socioeconomic status on physical and psychological health: lifestyle as a mediator. Int J Environ Res Public Health 16(2):281. 10.3390/ijerph16020281 30669511 10.3390/ijerph16020281 PMC6352250

[10] Singh GK, Jemal A (2017) Socioeconomic and racial/ethnic disparities in cancer mortality, incidence, and survival in the United States, 1950–2014: over six decades of changing patterns and widening inequalities. J Environ Public Health 2017:2819372. 10.1155/2017/2819372 28408935 10.1155/2017/2819372 PMC5376950

[11] Li S, He Y, Liu J et al (2024) An umbrella review of socioeconomic status and cancer. Nat Commun 15(1):9993. 10.1038/s41467-024-54444-2 39557933 10.1038/s41467-024-54444-2 PMC11574020

[12] Lundqvist A, Andersson E, Ahlberg I, Nilbert M, Gerdtham U (2016) Socioeconomic inequalities in breast cancer incidence and mortality in Europe—a systematic review and meta-analysis. Eur J Public Health 26(5):804–813. 10.1093/eurpub/ckw070 27221607 10.1093/eurpub/ckw070 PMC5054273

[13] Bhattacharyya O, Li Y, Fisher JL et al (2021) Low neighborhood socioeconomic status is associated with higher mortality and increased surgery utilization among metastatic breast cancer patients. The Breast 59:314–320. 10.1016/j.breast.2021.08.003 34388697 10.1016/j.breast.2021.08.003 PMC8361177

[14] Coughlin SS (2019) Social determinants of breast cancer risk, stage, and survival. Breast Cancer Res Treat 177(3):537–548. 10.1007/s10549-019-05340-7 31270761 10.1007/s10549-019-05340-7

[15] Kaur M, Patterson A, Molina-Vega J et al (2023) Area deprivation index in patients with invasive lobular carcinoma of the breast: associations with tumor characteristics and outcomes. Cancer Epidemiol Biomarkers Prev 32(8):1107–1113. 10.1158/1055-9965.EPI-22-1353 37257200 10.1158/1055-9965.EPI-22-1353 PMC10390860

[16] Yang LY, Yang LP, Zhu B (2017) Clinicopathological characteristics and survival outcomes of invasive lobular carcinoma in different races. Oncotarget 8(43):74287–74298. 10.18632/oncotarget.19396 29088785 10.18632/oncotarget.19396 PMC5650340

[17] Quinn RM, Bernal AM, Oh SY, Anampa JD (2024) Trends in incidence of invasive lobular carcinoma of the breast by race: patterns by age, cancer stage, and socioeconomic factors in the United States, 1992–2019. Clin Breast Cancer. 10.1016/j.clbc.2024.12.015 39837694 10.1016/j.clbc.2024.12.015 PMC12103291

[18] Boffa DJ, Rosen JE, Mallin K et al (2017) Using the national cancer database for outcomes research: a review. JAMA Oncol 3(12):1722–1728. 10.1001/jamaoncol.2016.6905 28241198 10.1001/jamaoncol.2016.6905

[19] Salem ME, Puccini A, Trufan SJ et al (2021) Impact of sociodemographic disparities and insurance status on survival of patients with early-onset colorectal cancer. Oncologist 26(10):e1730–e1741. 10.1002/onco.13908 34288237 10.1002/onco.13908 PMC8488791

[20] Deyo RA, Cherkin DC, Ciol MA (1992) Adapting a clinical comorbidity index for use with ICD-9-CM administrative databases. J Clin Epidemiol 45(6):613–619. 10.1016/0895-4356(92)90133-8 1607900 10.1016/0895-4356(92)90133-8

[21] Ciriello G, Gatza ML, Beck AH et al (2015) Comprehensive molecular portraits of invasive lobular breast cancer. Cell 163(2):506. 10.1016/j.cell.2015.09.033 26451490 10.1016/j.cell.2015.09.033 PMC4603750

[22] Michaut M, Chin SF, Majewski I et al (2016) Integration of genomic, transcriptomic and proteomic data identifies two biologically distinct subtypes of invasive lobular breast cancer. Sci Rep 6:18517. 10.1038/srep18517 26729235 10.1038/srep18517 PMC4700448

[23] McCart Reed AE, Lal S, Kutasovic JR et al (2019) LobSig is a multigene predictor of outcome in invasive lobular carcinoma. Npj Breast Cancer 5(1):1–11. 10.1038/s41523-019-0113-y 31263747 10.1038/s41523-019-0113-y PMC6597578

[24] Sadigh G, Gray RJ, Sparano JA et al (2022) Assessment of racial disparity in survival outcomes for early hormone receptor-positive breast cancer after adjusting for insurance status and neighborhood deprivation: a post hoc analysis of a randomized clinical trial. JAMA Oncol 8(4):579–586. 10.1001/jamaoncol.2021.7656 35175284 10.1001/jamaoncol.2021.7656 PMC8855314

[25] Parab AZ, Kong A, Lee TA et al (2024) Socioecologic factors and racial differences in breast cancer multigene prognostic scores in US women. JAMA Netw Open 7(4):e244862. 10.1001/jamanetworkopen.2024.4862 38568689 10.1001/jamanetworkopen.2024.4862 PMC10993076

[26] Hoskins KF, Calip GS, Huang HC, Ibraheem A, Danciu OC, Rauscher GH (2023) Association of social determinants and tumor biology with racial disparity in survival from early-stage. Hormone Depend Breast Cancer JAMA Oncol. 10.1001/jamaoncol.2022.7705 10.1001/jamaoncol.2022.7705 PMC9936381 36795405

[27] Molina Y, Silva A, Rauscher GH (2015) Racial/ethnic disparities in time to a breast cancer diagnosis: the mediating effects of healthcare facility factors. Med Care 53(10):872. 10.1097/MLR.0000000000000417 26366519 10.1097/MLR.0000000000000417 PMC4570266

[28] Rauscher GH, Allgood KL, Whitman S, Conant E (2012) Disparities in screening mammography services by race/ethnicity and health insurance. J Womens Health 21(2):154. 10.1089/jwh.2010.2415 10.1089/jwh.2010.2415 PMC3270049 21942866

[29] Linnenbringer E, Gehlert S, Geronimus AT, Linnenbringer E, Gehlert S, Geronimus AT (2017) Black-white disparities in breast cancer subtype: the intersection of socially patterned stress and genetic expression. AIMS Public Health 4(5):526–556. 10.3934/publichealth.2017.5.526 29333472 10.3934/publichealth.2017.5.526 PMC5764177

[30] Beckie TM (2012) A systematic review of allostatic load, health, and health disparities. Biol Res Nurs 14(4):311–346. 10.1177/1099800412455688 23007870 10.1177/1099800412455688

[31] Obeng-Gyasi S, Timsina L, Bhattacharyya O, Fisher CS, Haggstrom DA (2020) Breast cancer presentation, surgical management and mortality across the rural-urban continuum in the National Cancer Database. Ann Surg Oncol 27(6):1805–1815. 10.1245/s10434-020-08376-y 32206955 10.1245/s10434-020-08376-y

[32] Geronimus AT, Hicken M, Keene D, Bound J (2006) “Weathering” and age patterns of allostatic load scores among blacks and whites in the United States. Am J Public Health 96(5):826–833. 10.2105/AJPH.2004.060749 16380565 10.2105/AJPH.2004.060749 PMC1470581

[33] Reeves A, Elliott MR, Lewis TT, Karvonen-Gutierrez CA, Herman WH, Harlow SD (2022) Study selection bias and racial or ethnic disparities in estimated age at onset of cardiometabolic disease among midlife women in the US. JAMA Netw Open 5(11):e2240665. 10.1001/jamanetworkopen.2022.40665 36342714 10.1001/jamanetworkopen.2022.40665 PMC9641536

[34] Geronimus AT (1992) The weathering hypothesis and the health of African–American women and infants: evidence and speculations. Ethn Dis 2(3):207–221 1467758

[35] Goel N, Hernandez A, Cole SW (2024) Social genomic determinants of health: understanding the molecular pathways by which neighborhood disadvantage affects cancer outcomes. J Clin Oncol 42(30):3618–3627. 10.1200/JCO.23.02780 39178356 10.1200/JCO.23.02780 PMC12045328

[36] Miller GE, Chen E, Fok AK et al (2009) Low early-life social class leaves a biological residue manifested by decreased glucocorticoid and increased proinflammatory signaling. Proc Natl Acad Sci USA 106(34):14716–14721. 10.1073/pnas.0902971106 19617551 10.1073/pnas.0902971106 PMC2732821

[37] Miller GE, Chen E, Sze J et al (2008) A functional genomic fingerprint of chronic stress in humans: blunted glucocorticoid and increased NF-κB signaling. Biol Psychiatry 64(4):266–272. 10.1016/j.biopsych.2008.03.017 18440494 10.1016/j.biopsych.2008.03.017 PMC2581622

[38] Antoni MH, Bouchard LC, Jacobs JM et al (2016) Stress management, leukocyte transcriptional changes and breast cancer recurrence in a randomized trial: an exploratory analysis. Psychoneuroendocrinology 74:269–277. 10.1016/j.psyneuen.2016.09.012 27689900 10.1016/j.psyneuen.2016.09.012 PMC5159236

[39] Lee MJ, Rittschof CC, Greenlee AJ et al (2021) Transcriptomic analyses of black women in neighborhoods with high levels of violence. Psychoneuroendocrinology 127:105174. 10.1016/j.psyneuen.2021.105174 33647572 10.1016/j.psyneuen.2021.105174 PMC9191231

[40] Knight JM, Rizzo JD, Logan BR et al (2016) Low socioeconomic status, adverse gene expression profiles, and clinical outcomes in hematopoietic stem cell transplant recipients. Clin Cancer Res Off J Am Assoc Cancer Res 22(1):69–78. 10.1158/1078-0432.CCR-15-1344 10.1158/1078-0432.CCR-15-1344 PMC4703514 26286914

[41] Carlos RC, Obeng-Gyasi S, Cole SW et al (2022) Linking structural racism and discrimination and breast cancer outcomes: a social genomics approach. J Clin Oncol 40(13):1407–1413. 10.1200/JCO.21.02004 35108027 10.1200/JCO.21.02004 PMC9851699

[42] Barnard ME, Wang X, Petrick JL et al (2024) Psychosocial stressors and breast cancer gene expression in the Black Women’s Health Study. Breast Cancer Res Treat 204(2):327–340. 10.1007/s10549-023-07182-w 38127176 10.1007/s10549-023-07182-w PMC11232497

[43] Peek ME, Han JH (2004) Disparities in screening mammography: current status, interventions, and implications. J Gen Intern Med 19(2):184. 10.1111/j.1525-1497.2004.30254.x 15009798 10.1111/j.1525-1497.2004.30254.x PMC1492136

[44] Zanobini P, Bonaccorsi G, Giusti M et al (2023) Health literacy and breast cancer screening adherence: results from the population of Tuscany, Italy. Health Promot Int 38(6):daad77. 10.1093/heapro/daad177 10.1093/heapro/daad177 38146742

[45] Baccolini V, Isonne C, Salerno C et al (2022) The association between adherence to cancer screening programs and health literacy: A systematic review and meta-analysis. Prev Med 155:106927. 10.1016/j.ypmed.2021.106927 34954244 10.1016/j.ypmed.2021.106927

[46] Mobley LR, Tangka FKL, Berkowitz Z et al (2021) Geographic disparities in late-stage breast cancer diagnosis rates and their persistence over time. J Womens Health 30(6):807–815. 10.1089/jwh.2020.8728 10.1089/jwh.2020.8728 PMC8420944 33926216

[47] Sprague BL, Ahern TP, Herschorn SD, Sowden M, Weaver DL, Wood ME (2021) Identifying key barriers to effective breast cancer control in rural settings. Prev Med 152:106741. 10.1016/j.ypmed.2021.106741 34302837 10.1016/j.ypmed.2021.106741 PMC8545865

[48] Hillman S, Shantikumar S, Ridha A, Todkill D, Dale J (2020) Socioeconomic status and HRT prescribing: a study of practice-level data in England. Br J Gen Pract 70(700):e772. 10.3399/bjgp20X713045 32988956 10.3399/bjgp20X713045 PMC7523922

[49] Liu Y, Li C (2024) Hormone therapy and biological aging in postmenopausal women. JAMA Netw Open 7(8):e2430839. 10.1001/jamanetworkopen.2024.30839 39207753 10.1001/jamanetworkopen.2024.30839 PMC11362863

[50] Li CI, Weiss NS, Stanford JL, Daling JR (2000) Hormone replacement therapy in relation to risk of lobular and ductal breast carcinoma in middle-aged women. Cancer 88(11):2570–2577. 10.1002/1097-0142(20000601)88:11%3c2570::aid-cncr20%3e3.0.co;2-o 10861435 10.1002/1097-0142(20000601)88:11<2570::aid-cncr20>3.0.co;2-o

[51] Yang DX, Khera R, Miccio JA et al (2021) Prevalence of missing data in the national cancer database and association with overall survival. JAMA Netw Open 4(3):e211793. 10.1001/jamanetworkopen.2021.1793 33755165 10.1001/jamanetworkopen.2021.1793 PMC7988369

[52] Christgen M, Cserni G, Floris G et al (2021) Lobular breast cancer: histomorphology and different concepts of a special spectrum of tumors. Cancers 13(15):3695. 10.3390/cancers13153695 34359596 10.3390/cancers13153695 PMC8345067

